# Feasibility and safety of virtual-reality-based early neurocognitive stimulation in critically ill patients

**DOI:** 10.1186/s13613-017-0303-4

**Published:** 2017-08-02

**Authors:** Marc Turon, Sol Fernandez-Gonzalo, Mercè Jodar, Gemma Gomà, Jaume Montanya, David Hernando, Raquel Bailón, Candelaria de Haro, Victor Gomez-Simon, Josefina Lopez-Aguilar, Rudys Magrans, Melcior Martinez-Perez, Joan Carles Oliva, Lluís Blanch

**Affiliations:** 1 Centro de Investigación Biomédica En Red en Enfermedades Respiratorias (CIBERES), Instituto de Salud Carlos III, Madrid, Spain; 2 Institut d’Investigació i Innovació Parc Taulí (I3PT), Fundació Parc Taulí, Sabadell, Spain; 3 Department of Clinical and Health Psychology, Universitat Autònoma de Barcelona, International Excellence Campus, Bellaterra, Spain; 4 Centro de Investigación Biomédica En Red en Salud Mental (CIBERSAM), Instituto de Salud Carlos III, Madrid, Spain; 5 Neurology Department, Parc Taulí Hospital Universitari, Institut d’Investigació i Innovació Parc Taulí (I3PT), Universitat Autònoma de Barcelona, Sabadell, Spain; 6 Critical Care Department, Parc Taulí Hospital Universitari, Institut d’Investigació i Innovació Parc Taulí (I3PT), Universitat Autònoma de Barcelona, Sabadell, Spain; 7 BSICOS Group, 13A, Universidad de Zaragoza&CIBER-BBN, Saragossa, Spain

**Keywords:** Neurocognitive impairments, Critically ill patients, Neurocognitive stimulation, Virtual reality, Early intervention, ICU

## Abstract

**Background:**

Growing evidence suggests that critical illness often results in significant long-term neurocognitive impairments in one-third of survivors. Although these neurocognitive impairments are long-lasting and devastating for survivors, rehabilitation rarely occurs during or after critical illness. Our aim is to describe an early neurocognitive stimulation intervention based on virtual reality for patients who are critically ill and to present the results of a proof-of-concept study testing the feasibility, safety, and suitability of this intervention.

**Methods:**

Twenty critically ill adult patients undergoing or having undergone mechanical ventilation for ≥24 h received daily 20-min neurocognitive stimulation sessions when awake and alert during their ICU stay. The difficulty of the exercises included in the sessions progressively increased over successive sessions. Physiological data were recorded before, during, and after each session. Safety was assessed through heart rate, peripheral oxygen saturation, and respiratory rate. Heart rate variability analysis, an indirect measure of autonomic activity sensitive to cognitive demands, was used to assess the efficacy of the exercises in stimulating attention and working memory.

**Results:**

Patients successfully completed the sessions on most days. No sessions were stopped early for safety concerns, and no adverse events occurred. Heart rate variability analysis showed that the exercises stimulated attention and working memory. Critically ill patients considered the sessions enjoyable and relaxing without being overly fatiguing.

**Conclusions:**

The results in this proof-of-concept study suggest that a virtual-reality-based neurocognitive intervention is feasible, safe, and tolerable, stimulating cognitive functions and satisfying critically ill patients. Future studies will evaluate the impact of interventions on neurocognitive outcomes.

*Trial registration* Clinical trials.gov identifier: NCT02078206

**Electronic supplementary material:**

The online version of this article (doi:10.1186/s13613-017-0303-4) contains supplementary material, which is available to authorized users.

## Background

Growing evidence suggests that critical illness often results in significant long-term morbidities. One-third of patients in intensive care units (ICU) develop neurocognitive impairments in a magnitude similar to mild–moderate dementia [1] persisting for years after hospital discharge [2, 3]. ICU-related neurocognitive impairments are particularly pronounced in regard to memory, executive functions, attentional functions, and processing speed [1, 3–10]. These impairments have far-reaching consequences and adversely impact patients’ lives, contributing to impaired ability to perform activities of daily living, to decreased quality of life for patients and relatives, to increased medical costs, and to inability to return to work [11, 12]; however, patients rarely undergo rehabilitation during or after critical illness.

Rehabilitation strategies during the ICU stay have mainly involved physical interventions such as early mobilizations [13–16] to enhance functional recovery. Other strategies include early detection and treatment of delirium [17], and occupational therapy [18]. Recently, early rehabilitation strategies in the ICU have been extended beyond physical therapy to include cognitive interventions [19].

Physical and cognitive rehabilitation tools based on new technologies are being used successfully in patients with dementia, traumatic brain injury, stroke [20], and schizophrenia [21]. However, critically ill patients are usually bedridden and often unable to communicate verbally, posing special challenges for the development and use of technologies for cognitive intervention. Thus, we sought to design a virtual-reality-based intervention that could be used to provide critically ill patients with neurocognitive stimulation early in the ICU stay.

Another challenge in this context is determining whether an intervention actually results in brain stimulation. Heart rate variability (HRV), a surrogate marker of sympathetic and vagal activity, is a particularly sensitive index of the changes in the neural network in response to cognitive requirements. The magnitude of the changes in HRV is inversely related to the individual level of cognitive effort during cognitive tasks. HRV decreases during tasks involving specifically selective and maintained attention [22, 23] and executive functions [24, 25]. Thus, HRV analysis seems useful to confirm that neurocognitive intervention results in brain stimulation in critically ill patients.

This paper describes a virtual-reality-based early neurocognitive stimulation intervention in critically ill patients and presents preliminary results of a proof-of-concept study to assess the feasibility and safety of the intervention, its tolerability in terms of difficulty and cognitive load, and the effectiveness of the cognitive exercises in stimulating the brain. Some of these results have been presented in abstract form [26–28].

## Methods

### Inpatient early neurocognitive intervention

**Fig. 1:**
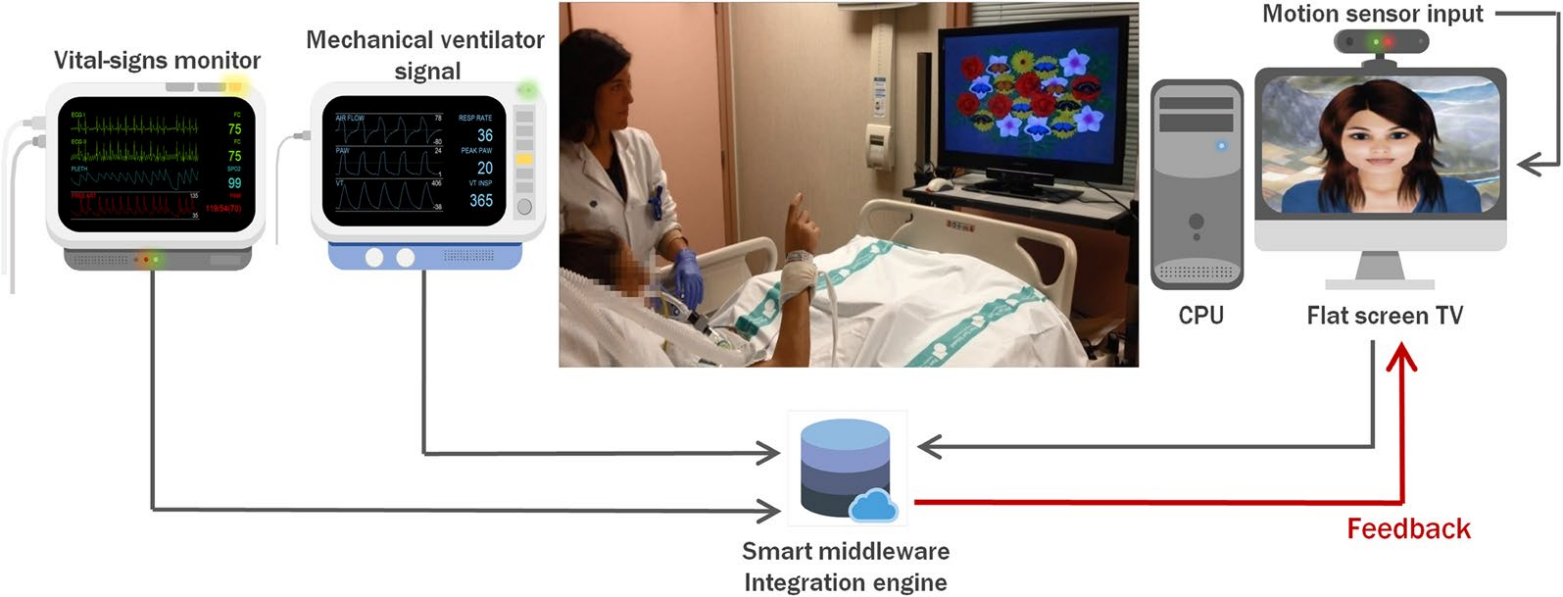
Schematic diagram of the smart middleware connecting ENRIC platform, bedside monitor and ventilator (screenshots of bedside monitor and ventilator courtesy of BetterCare^®^, Barcelona, Spain). The platform uses smart middleware to connect the cognitive stimulation software with input from a motion sensor (Kinect^®^, Microsoft, Redmond, Washington, USA) that captures and interprets patient movements. The platform also incorporates a system to collect and store physiological data (BetterCare^®^, Barcelona, Spain) from bedside monitors and ventilators that could be used to adapt the level of cognitive stimulation to patients’ clinical condition

An interdisciplinary team of neuropsychologists, nurses, critical care physicians, and biomedical engineers designed the *Early Neurocognitive Rehabilitation in Intensive Care* (ENRIC) platform for neurocognitive stimulation in critically ill patients. This platform uses stimulation software with low cognitive load exercises specifically designed or adapted for critically ill patients. The stimulation software is based on virtual reality techniques immersing patients in a relaxing environment; a virtual avatar accompanies patients, orienting them in time, delivering instructions, motivating them to complete exercises, and encouraging them to relax. Different exercises based on exercises proven effective in cognitive rehabilitation programs for acquired brain injury focus on attention and executive functions [20]. Details about the stimulation software and videos are reported in the Additional files 1 and 2. The ENRIC platform consists of a central processing unit, a flat-screen TV, and a motion sensor (Kinect^®^ Microsoft, Redmond, Washington, USA) placed on a medical cart to allow it to be moved easily to each ICU bed. The system captures and interprets patient movements through smart middleware connecting the cognitive stimulation software with input from the motion sensor. This approach to patient–system interaction avoids the risk of cross-infections inherent in sharing physical devices such as joysticks and touch tablets. The platform also incorporates a system to collect and store physiological data (BetterCare^®^, Barcelona, Spain) from bedside monitors and ventilators [29] that could be used to adapt the level of cognitive stimulation to patients’ clinical condition (Fig. 1). The ENRIC platform turns on when it is plugged in. Before each session, it is necessary to calibrate the Kinect^®^ motion sensor and to adjust the distance between the patient and sensor; patients are asked to raise their hands in front of the sensor to check detection and motion recognition. Sometimes, the ICU bed needs to be raised or lowered. Bed adjustments and sensor calibration take less than 2 min. Although patients have no physical contact with any components of the device at any time, for reasons of infection control, the device is cleaned and disinfected after each session when it is used in isolated patients.

### Patients

Patients were recruited from a mixed medical/surgical ICU with 16 beds in single rooms at a university teaching hospital. Patients aged 18–85 years old who were undergoing or had undergone invasive mechanical ventilation for ≥24 h were eligible. Patients with a history of neurologic disease, dementia, focal brain injury at ICU admission, serious psychiatric disorders, or mental retardation were excluded. Deaf and/or blind patients were also excluded. Eligible patients were screened daily and invited to participate when they achieved minimum levels of consciousness to interact with the ENRIC platform [Glasgow Coma Scale (GCS) ≥13] and sedation (Richmond Agitation–Sedation Scale (RASS) −1 to +1).

### Procedure

Demographic data and severity (Acute Physiologic and Chronic Health Evaluation II (APACHE-II) and Sequential Organ Failure Assessment (SOFA) scores) were recorded at inclusion. Level of consciousness was assessed daily from ICU admission to initiation of the intervention. Upon reaching GCS ≥13, patients were screened daily for delirium using the Spanish version of the Confusional Assessment Method for the Intensive Care Unit (CAM-ICU) and for level of alertness using the RASS.

 Patients received the neurocognitive stimulation sessions each day in their own beds when they were alert and calm (RASS −1 to +1) until discharge from the ICU. Sessions were guided by a neuropsychologist and supervised by an ICU nurse. Sessions aimed to provide cognitive stimulation and engagement through exercises, not necessarily obtaining correct answers. The exercises included in each session were determined by patients’ alertness level and ability to raise each arm separately with elbow straight against gravity (see Additional file 3: Figure S1 for a detailed description of the protocol). The clinical team predefined the length of the session as a minimum of 15 and maximum of 20 min. However, whenever clinical conditions allowed it, patients were encouraged to continue as long as they could without fatigue. Physiological data were recorded 20 min before sessions, during sessions, and after sessions.

Before this proof-of-concept study, six healthy volunteers tested the ENRIC platform in a session simulating ICU patients’ situation and then rated their experience on a five-point Likert scale. The first six patients included in the study also completed the same ad hoc survey about their experience with the ENRIC platform. Finally, ICU personnel, including nurses, physicians, and physiotherapists, were asked to complete an acceptance survey, giving their opinions about the compatibility of the ENRIC platform with ICU workload (see Additional file 4).

### Measures

Feasibility was assessed in terms of the practicability of delivering neurocognitive stimulation early during critical illness: rates of patients eligible to receive the intervention and of patients who underwent the intervention, number of sessions performed, timing of initiation, and duration of each session.

Safety was assessed by considering the sessions that had to be stopped early for clinical reasons, through analyses of heart rate (HR), peripheral oxygen saturation (SpO_2_), and respiratory rate (RR). The safety analysis was based on expert consensus criteria for unsafe active mobilization of mechanically ventilated critically ill patients [30]: SpO_2_ < 90%, RR > 35 breaths per minute, and HR > 150 beats per minute, all during ≥5 min. When out-of-range values were present at the beginning of the session, changes >20% from baseline in any physiological parameter were also considered unsafe events requiring the session to be stopped.

Tolerability was assessed in terms of the extent to which sessions were completed and to which patients tolerated the difficulty and cognitive load of the exercises included. Thus, we recorded number of interrupted sessions and cause of interruption, as well as the type of exercises in each session.

The effectiveness in stimulating the brain was assessed in terms of autonomic reactivity during sessions using HRV frequency-domain analyses [31]. Spectral analysis was performed over consecutive 2-min R–R intervals series with an overlap of 1 min to ensure an analysis interval as stationary as possible. HRV was derived from the detected normal beat occurrence time series using the integral pulse frequency modulation (IPFM) model, which takes ectopic beats into accounts [32]. Since the mean heart rate is not constant (it evolves continuously in challenging situations, such as physical and mental effort, especially in critically ill patients), the HRV signal was corrected by the time-varying mean heart rate [33], yielding the modulating signal which, according to the IPFM model, represents autonomic nervous system modulation over the sinoatrial node. The modulating signal was sampled at 4 Hz, and its power spectral density was estimated. Low-frequency (LF) power and high-frequency (HF) power were computed by integrating the area under the power spectral density in the LF and HF bands, respectively. The LF band was 0.04–0.15 Hz. The HF band was centered on the respiratory frequency, and bandwidth ranged from 0.15 to 0.3 Hz in function of respiratory stability. We also included total power, an index of overall autonomic modulation summing the power in the LF and HF bands. LF values were normalized (LFn) by total power to exclusively reflect sympathetic modulation [31] and were expressed in normalized units, while HF values were expressed in adimensional units to reflect parasympathetic activity.

### Statistical analysis

Mean scores on the satisfaction survey were computed from the individual raw scores for all items included in each category after converting scores of negatively worded items to positives. Mean scores were analyzed using the Mann–Whitney *U* test.

No formal power calculations were undertaken; rather sufficient participants were recruited during ~9 months to determine factors such as recruitment rates in relation to feasibility outcomes.

Feasibility, safety, and tolerability outcomes were analyzed using descriptive statistics. To analyze whether the intervention actually stimulated the brain, LFn, HF, and total power indices during the sessions were compared with baseline values using the Wilcoxon signed-rank test, because values for these variables were not normally distributed. Because patients received the total range of neurocognitive exercises during the first three sessions, to avoid potential effects on autonomic reactivity due to the lack of novelty in subsequent sessions, HRV analysis was limited to the first three sessions.

All analyses were performed with SPSS version 19.0 (IBM, Armonk, NY); significance was set at 0.05. Results are presented as means and ranges or n/N (%), unless otherwise noted.

## Results

### Patients’ clinical and sociodemographic characteristics

**Fig. 2:**
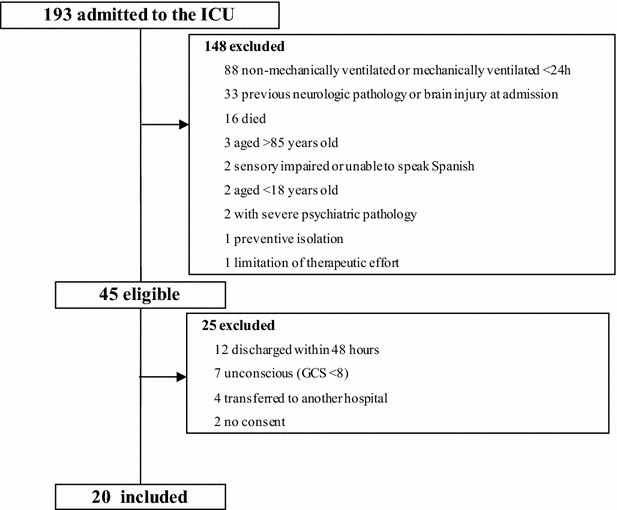
Flow diagram of sample. During a period comprising ~9 months, 193 patients were admitted to the ICU; 148 met at least one exclusion criterion. Of the 45 eligible patients, 25 were finally excluded. Thus, 20 patients received the early neurocognitive intervention

Between April 2014 and December 2014, 193 patients were admitted to the ICU; 148 met at least one exclusion criterion. Of the 45 eligible patients, 25 were excluded for the following reasons: 12 were discharged within 48 h, 7 remained unconscious, 4 were transferred to other centers, and 2 did not consent (Fig. 2). Thus, 20 patients received the early neurocognitive intervention; Table 1 summarizes their clinical and sociodemographic characteristics.

**Table 1:** Clinical and sociodemographic characteristics of critically ill patients undergoing early neurocognitive intervention (n = 20)

Age, years (M, SD)	65	10
Sex (N, %)		
Male	14	63.66
Female	6	27.33
Diagnosis (N, %)		
Medical		
Pneumonia	3	15
Septic shock	3	15
Acute respiratory failure	1	5
ARDS	1	5
Pancreatitis	1	5
Toxic intake	1	5
Unplanned surgery		
Peritonitis	3	15
Multiple trauma	2	10
Intestinal perforation	2	10
Pneumoperitoneum	1	5
Planned surgery		
Hemorrhagic shock	1	5
Esophageal perforation	1	5
Receiving MV at inclusion (N, %)	7	35
APACHE-II (M, SD)	24.84	9.04
SOFA (M, SD)	9.58	4.23
GCS (M, SD)	10.33	5.91
ICU LoS, days (Md, IQR)	16.00	2.00
Total MV time, days (Md, IQR)	9.50	1.00
Sedation, days (Md, IQR)	5.00	0.00
Delirium, days (Md, IQR)	1.39	0.80
Septic shock (N, %)	12	60
Cardiac arrest (N, %)	1	5

*APACHE*-*II* Acute Physiology and Chronic Health Evaluation II, *ARDS* acute respiratory distress syndrome, *GCS* Glasgow Coma Scale, *ICU* intensive care unit, *IQR* interquartile range, *LoS* length of stay, *M* mean, *Md* median, *MV* mechanical ventilation, *SD* standard deviation

### Feasibility of early neurocognitive stimulation

All 20 patients (100%) received the early neurocognitive intervention on at least one study day. The first session was delivered 10.0 (2.0–23.0) days after ICU admission. Once patients started the intervention, sessions were delivered on 74.3% (25.0–100.0%) of the eligible study days until ICU discharge.

During the study, 76 sessions were delivered [mean number of sessions per patient, 3.8 (1.0–8.0); mean duration of each session, 17.5 (12.0–31.0) minutes].

### Safety of early neurocognitive stimulation

Table 2a, b summarizes HR, RR, and SpO_2_ at baseline, during the session, and after the session and indicates the percentage of patients who were mechanically ventilated during each session. No unsafe HR values were observed in recordings at baseline or during the session; after the first session, unsafe HR values lasting less than 5 min were observed in one patient (1/20, 5%) after the first session and in another patient (1/11, 9.1%) after the fourth. During sessions, some patients reached unsafe minimum SpO_2_ values lasting less than 5 min (session #1: 2/20, 10%; session #2: 1/17, 5.9%; session #3: 1/13, 7.7%; and session #4: 1/11, 9.1%), and some patients reached unsafe RR (session #1: 6/20, 30%; session #2: 11.8%; session #3: 2/13, 15.4%; session #4: 3/11, 27.3%; session #5: 4/8, 50%; session #6: 1/4, 25%; and session #7: 1/2, 50%). Nevertheless, no sessions were stopped early for safety concerns, and no adverse events (e.g., inadvertent removal of catheters or endotracheal tubes) occurred. No changes in physiological parameters ≥20% from baseline were observed during the sessions. No patients received vasoactive agents during sessions.

**Table 2:** Values of physiological parameters at baseline, during session, and post-session

	Session 1			Session 2			Session 3			Session 4		
	(n = 20; MV in 35%)			(n = 17; MV in 35%)			(n = 13; MV in 38%)			(n = 11; MV in 36%)		
	Baseline	Session	Post	Baseline	Session	Post	Baseline	Session	Post	Baseline	Session	Post
(a)												
HR, Md (IQR)	92 (14)	92.5 (15.75)	91 (10.5)	82 (23)	84 (21.25)	81.5 (25.5)	96 (19)	90 (15)	90 (15)	86 (31.75)	67 (34.5)	90.5 (35.5)
Range	67–120	70–119	70–153^a^	56–127	68–126	65–125	69–134	72–137	72–137	62–128	59–129	67–168^a^
SPO_2_, Md (IQR)	96 (5)	95.5 (4.25)	95 (5)	94.5 (3.75)	94.5 (4.5)	94 (3.75)	96 (4)	96 (2)	96 (2)	97 (2.75)	97 (2.75)	97 (2.5)
Range	88^b^–100	89^b^–100	90–100	89^b^–100	89^b^–100	89^b^–100	90–100	89^b^–100	90–100	91–100	89^b^–100	90–100
RR, Md (IQR)	22 (13)	24 (12)	22 (13.5)	22.5 (12.25)	23.5 (11)	24 (10.75)	25 (14)	25 (9)	22 (8)	20.5 (16.25)	21.5 (11.5)	24.5 (15)
Range	11–54^c^	10–41^c^	10–54^c^	9–46^c^	9–40^c^	9–44^c^	12–47^c^	12–37^c^	12–41^c^	12–42^c^	12–41^c^	9–43^c^

	Session 5			Session 6			Session 7			Session 8		
	(n = 8; MV in 50%)			(n = 4; MV in 50%)			(n = 2; MV in 100%)			(n = 1; MV in 100%)		
	Baseline	Session	Post	Baseline	Session	Post	Baseline	Session	Post	Baseline	Session	Post
(b)												
HR, Md (IQR)	102 (25.5)	103 (27.5)	101 (24)	108 (12)	110 (16)	111 (8.5)	112.5 (4.75)	115 (1.25)	111 (1)	126 (4)	123 (2)	127 (3)
Range	61–139	63–138	64–128	79–118	78–121	88–120	104–123	93–123	107–114	121–134	119–125	117–134
SPO_2_, Md (IQR)	98 (2)	97 (1.5)	97 (2.5)	97 (4)	97 (2.5)	98 (2)	99.5 (1.25)	98.5 (1.25)	98 (1)	98 (1)	98 (0)	99 (1)
Range	89^b^–100	91–100	92–100	90–100	91–100	90–100	97–100	95–100	95–100	94–99	98–99	96–100
RR, Md (IQR)	21 (15)	23 (13)	23 (15.5)	25 (6)	25 (6.5)	26 (6)	26.5 (7)	27.5 (6.5)	29 (2)	27 (3)	26 (3)	25 (6)
Range	11–40^c^	10–39^c^	10–50^c^	13–38^c^	13–36^c^	13–37^c^	19–40^c^	18–37^c^	18–39^c^	14–33	20–33	12–40^c^

*MV* mechanical ventilation, *Md* median, *IQR* interquartile range, *HR* heart rate, *SpO*_*2*_ peripheral oxygen saturation, *RR* respiratory rate

^a^HR ≥ 150 bpm

^b^SpO_2_ ≤ 90%

^c^RR ≥ 35 breaths/min

### Tolerability of early neurocognitive stimulation

Patients completed 66/76 (86.8%) of possible neurocognitive stimulation sessions. Most (70%) incomplete sessions occurred on the first day. Reasons for discontinuing a session were fatigue (50%), extreme sleepiness (20%), competing medical procedures (20%), and confusion (10%). Discontinuation did not preclude participation in subsequent sessions.

**Fig. 3:**
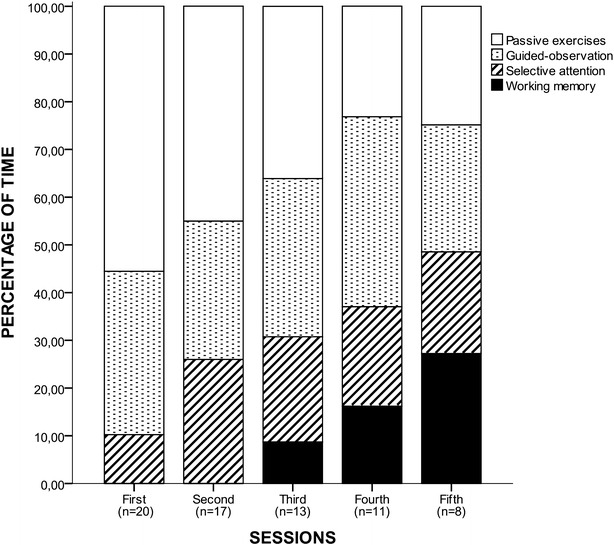
Time distribution (%) of neurocognitive exercises for each session during the first five sessions. In the first session, *passive exercises* requiring simple attention and gross motor functions were the most performed exercises. In subsequent sessions, the time spent on *passive exercises* gradually decreased, while the time spent on exercises focusing on *selective attention* and *working memory* increased. *Guided*-*observation exercises* were well tolerated for patients from the first session. The most complex exercises, focusing on *working memory*, were only performed from the third session, and the time assigned for these exercises was increased progressively with each session. *Note* that in the fifth session, equal time was assigned to each type of exercises

Figure 3 illustrates the composition of the first five sessions, with progressively more challenging exercises. In the first session, *passive exercises* requiring simple attention and gross motor functions accounted for 55.6% of the total time. In subsequent sessions, the time spent on *passive exercises* gradually decreased, while the time spent on exercises requiring more complex cognitive effort (*selective attention* and *working memory exercises*) increased. Patients correctly followed the neuropsychologist’s orders in the *guided*-*observation exercises*, which represented 34.2% of the total session time. *Selective attention exercises* accounted for 10.2% of the total time. The most complex exercises, focusing on *working memory*, were only performed from the third session, and the time assigned for these exercises was increased progressively with each session. In the fifth session, equal time was assigned to each type of exercises.

### Effectiveness of neurocognitive exercises in stimulating the brain

Table 3 displays the results of HRV analysis for the three main frequency-domain indices. In session #1, LFn was lower (*p* = 0.02) than baseline values. In session #2, all indices were lower than baseline (LFn, *p* = 0.01; HF, *p* = 0.03; and total power, *p* = 0.01). In session #3, total power was lower than baseline (*p* = 0.02).

**Table 3:** Heart rate variability indices at baseline and during session

	Session 1		*p*	Session 2		*p*	Session 3		*p*
	Baseline	Session		Baseline	Session		Baseline	Session	
LFn, Md (IQR)	3.87 × 10^−5^ (2.08 × 10^−4^)	3.02 × 10^−5^ (8.64 × 10^−5^)	0.02	7.44 × 10^−5^ (4.68 × 10^−4^)	6.91 × 10^−5^ (3.79 × 10^−4^)	0.01	9.17 × 10^−5^ (5.31 × 10^−4^)	6.74 × 10^−5^ (3.85 × 10^−4^)	0.23
HF, Md (IQR)	1.63 × 10^−3^ (1.64 × 10^−1^)	6.48 × 10^−5^ (2.31 × 10^−1^)	0.65	2.84 × 10^−5^ (5.17 × 10^−5^)	2.64 × 10^−5^ (2.05 × 10^−4^)	0.03	6.94 × 10^−5^ (1.21 × 10^−3^)	6.19 × 10^−5^ (8.71 × 10^−4^)	0.11
Total, Md (IQR)	2.88 × 10^−3^ (1.64 × 10^−1^)	2.25 × 10^−4^ (2.32 × 10^−1^)	0.38	9.85 × 10^−5^ (4.31 × 10^−4^)	9.81 × 10^−5^ (6.43 × 10^−4^)	0.01	2.44 × 10^−4^ (1.46 × 10^−3^)	1.08 × 10^−4^ (2.26 × 10^−3^)	0.02

*LFn* power in low-frequency band expressed in normalized units, *HF* power in high-frequency band expressed in adimensional units, *Total* total power expressed in adimensional units, *Md* median, *IQR* interquartile range

*p* values for nonparametric Wilcoxon signed-rank test

### Satisfaction and acceptance survey results

Table S1 compares satisfaction survey scores between healthy volunteers and critically ill patients (see Additional file 4). Patients scored higher than volunteers for *relaxation* (*p* = 0.002) and *system interaction* (*p* = 0.004) and lower for *boredom* (*p* = 0.041). Additional file 4: Table S2 reports the scores on the acceptance survey for ICU personnel, including the overall mean as well as mean scores for physicians, nurses, and physiotherapists. The lowest rated item was *Compatibility with physical infrastructure and ICU facilities*, and the highest rated items were *Compatibility with pharmacologic treatment* and *Compatibility with physiotherapy*.

## Discussion

Our results indicate that this virtual-reality-based neurocognitive intervention is feasible, safe, and tolerable for critically ill patients (including mechanically ventilated patients) and that the exercises used provide cognitive stimulation. The satisfaction and acceptance surveys found that critically ill patients considered the sessions enjoyable and relaxing without being overly fatiguing. Furthermore, ICU personnel considered the neurocognitive intervention compatible with the routine workload and the usual treatments prescribed in the ICU.

Neurocognitive impairments in ICU survivors may be understood as a manifestation of occult brain damage resulting from pathophysiological mechanisms related to critical illness and crosstalk between organs [34, 35]. Therefore, it might be interesting to consider ICU patients as a subset of brain-injured patients and treat them with tools such as cognitive stimulation that have proven effective in treating neurocognitive impairments in outpatient populations [36]. However, neurocognitive stimulation techniques commonly used in post-acute patients are often unfeasible in critically ill patients, who may be bedridden, have low awareness levels that fluctuate during the day, have reduced mobility due to myopathy and ICU-acquired weakness, and are unable to communicate verbally because they are receiving mechanical ventilation. To overcome these barriers to implementing neurocognitive interventions in the ICU, clinicians need safe, feasible, and easy-to-use tools for neurocognitive stimulation. To our knowledge, this study is the first to establish a proof of concept for virtual-reality-based neurocognitive stimulation in critically ill patients.

The motion sensor in the ENRIC platform provides a feasible solution to mobility and communication problems in early neurocognitive stimulation for critically ill patients. Brummel et al. [19] started an intervention combining physical therapy and cognitive therapy within 72 h after ICU admission when patients achieved RASS −3 to −2, but their cognitive protocol started only when they achieved RASS −1 to +1. In our study, neurocognitive stimulation started when patients reached RASS −1 to +1 (median, 10 days after ICU admission; range 2–23 days), suggesting that deep-sedation strategies, in addition to leading to increased prevalence of brain dysfunction in ICU [37], are the most important barrier to this kind of interventions in the ICU. In a pilot study of feasibility of physical interventions in critically ill patients, profound levels of sedation made 27% of physical sessions impossible [38]; protocols reducing sedation could help enable both physical and neurocognitive interventions in the ICU. Nonetheless, patients in our study successfully received neurocognitive stimulation during 74.3% of their time in the ICU, reflecting a wide therapeutic window for treatment.

No sessions were stopped early for safety concerns, and no adverse events (e.g., inadvertent removal of catheters or endotracheal tubes) occurred. Patients’ tolerance to increasingly difficult exercises in neurocognitive stimulation sessions increased as patients’ physical and cognitive status improved, showing that the intervention is safely adaptable to each patient’s physical and cognitive condition from the first day to ICU discharge.

Importantly, the intervention seems effective in stimulating the brain. Our HRV analysis found an overall decrease in the spectral components of HRV during the neurocognitive stimulation intervention. Since HRV decreases when attentional [22, 23] and executive functions [24, 25] are stimulated, these results indicate that the intervention stimulated these cognitive functions in our critically ill patients, including those with low alertness levels.

There is some evidence that neurocognitive interventions can help mitigate long-term ICU-related neurocognitive deficits. Jackson et al. [39] found that combined cognitive and physical therapy improved executive function and instrumental activities of daily living in a sample of ICU survivors. However, delaying interventions until after ICU discharge may be less effective, and introducing interventions only when cognitive and physical decline has already appeared seems insufficient to completely reverse deficits [40]. Early neurocognitive stimulation promises to improve outcomes in critically ill patients, potentially decreasing the incidence and duration of delirium, shortening ICU and hospital stays, and ultimately reducing costs while improving cognitive function and quality of life after discharge. Nevertheless, neurocognitive interventions are rarely routinely delivered during hospitalization. Our small sample from a single center limits the generalizability of our specific findings. However, this proof-of-concept study aimed only to assess feasibility of neurocognitive stimulation in the ICU. Therefore, many key questions were not addressed (e.g., the best type of intervention or exercises for cognitive stimulation, the optimal time to start neurocognitive interventions, the optimal duration and intensity of interventions, and impact of interventions on long-term outcomes). These questions should be assessed in larger samples. Ultimately, HRV can be influenced by certain drugs typically prescribed in ICU, among which include anticholinergic drugs, agents with effect on adrenergic receptors, angiotensin-converting enzyme inhibitors, or even calcium channel blockers, so the effect should be considered cautiously while interpreting HRV results.

## Conclusions

This proof-of-concept study shows that virtual-reality-based neurocognitive interventions are feasible, safe, and tolerable for critically ill patients. It also shows that the ENRIC platform stimulates cognitive functions and was well received by patients. These preliminary results lay the groundwork for implementing such interventions on a larger scale to evaluate their efficacy.

## Additional files

**Additional file 1: Video 1.** Neurocognitive stimulation session with mechanically ventilated patient.

**Additional file 2: Video 2.** Details of neurocognitive stimulation software and system interaction.

**Additional file 3: Figure S1.** Diagram of sessions planning based on alertness and mobility parameters. RASS score and the ability to raise autonomously each arm separately with their elbow straight against gravity determined the intensity of stimulation and which exercises to include in each session.

**Additional file 4: Table S1.** Satisfaction survey scores in healthy volunteers versus critically ill patients. **Table S2.** Acceptance survey scores in physicians, nurses, and physiotherapists.

## Abbreviations

APACHE-II: Acute Physiologic and Chronic Health Evaluation II
CAM-ICU: Confusional Assessment Method for ICU
ENRIC: Early Neurocognitive Rehabilitation in Intensive Care
GCS: Glasgow Coma Scale
HF: high frequency
HR: heart rate
HRV: heart rate variability
ICU: intensive care unit
IPFM: integral pulse frequency modulation
LF: low frequency
LFn: normalized low frequency
RASS: Richmond Agitation–Sedation Scale
RR: respiratory rate
SOFA: Sequential Organ Failure Assessment
SpO_2_: oxygen peripheral saturation
