# Supplementary figures and images for: Feasibility and safety of virtual-reality-based early neurocognitive stimulation in critically ill patients
Source: Ann Intensive Care. 2017 Aug 2;7:81. doi: 10.1186/s13613-017-0303-4 (PMC5540744; doi:10.1186/s13613-017-0303-4)

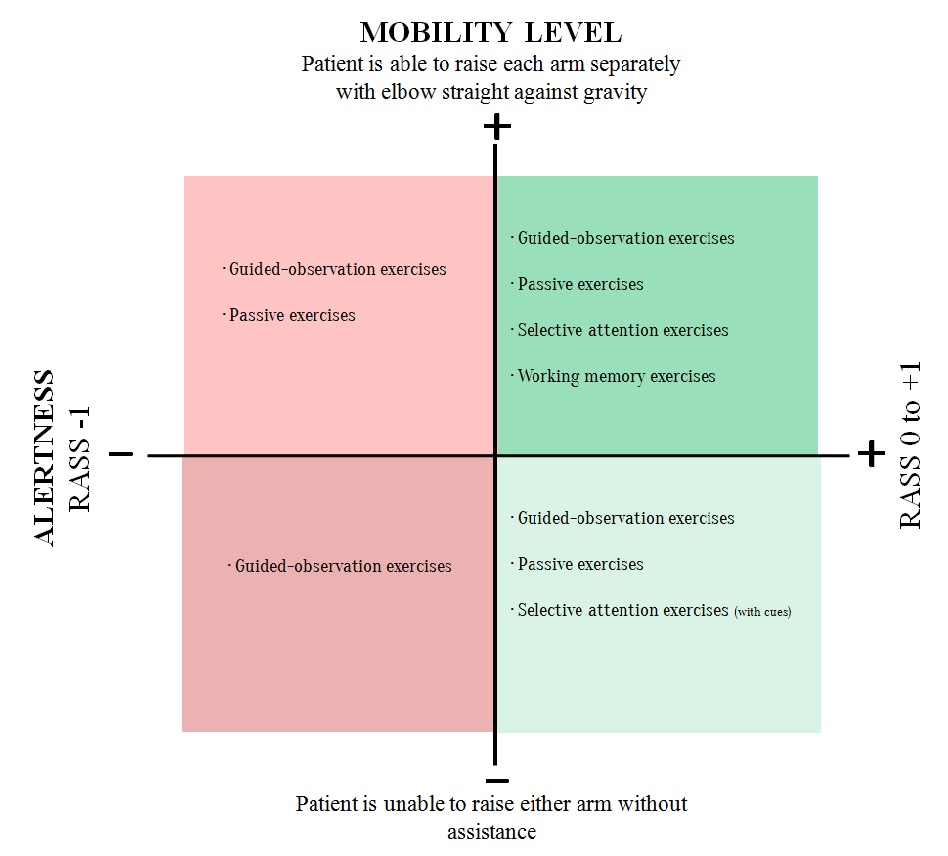

Supplement: Supplementary file 3 — Additional file 3: Figure S1. Diagram of sessions planning based on alertness and mobility parameters. RASS score and the ability to raise autonomously each arm separately with their elbow straight against gravity determined the intensity of stimulation and which exercises to include in each session. [file 13613_2017_303_MOESM3_ESM.doc]
