# Supplementary material for: Feasibility and safety of virtual-reality-based early neurocognitive stimulation in critically ill patients
Source: Ann Intensive Care. 2017 Aug 2;7:81. doi: 10.1186/s13613-017-0303-4 (PMC5540744; doi:10.1186/s13613-017-0303-4)
Supplement: Supplementary file 4 — Additional file 4: Table S1. Satisfaction survey scores in healthy volunteers versus critically ill patients. Table S2. Acceptance survey scores in physicians, nurses, and physiotherapists. [file 13613_2017_303_MOESM4_ESM.doc]

**Electronic Supplementary Material**

**Feasibility and safety of virtual-reality-based early neurocognitive stimulation in critically ill patients**

Marc Turon^1,2,3*^, Sol Fernandez-Gonzalo^2,4^, Mercè Jodar^3,4,5^, Gemma Gomà^2,6^, Jaume Montanya^1^, David Hernando^7^, Raquel Bailón^7^ , Candelaria de Haro^6^, Victor Gomez-Simon^2,6^, Josefina Lopez-Aguilar^1,2^, Rudys Magrans^1,2^, Melcior Martinez-Perez^1,6^, Joan Carles Oliva^2^, Lluís Blanch^1,2,6^

^1^Centro de Investigación Biomédica En Red en Enfermedades Respiratorias (CIBERES), Instituto de Salud Carlos III. Madrid, Spain

^2^Research Department, Institut d’Investigació i Innovació Sanitària Parc Taulí (I3PT), Fundació Parc Taulí, Corporació Sanitària Universitària Parc Taulí, Sabadell, Spain

^3^Department of Clinical and Health Psychology, Universitat Autònoma de Barcelona, International Excellence Campus 08193, Bellaterra, Spain

^4^Centro de Investigación Biomédica En Red en Salud Mental (CIBERSAM), Instituto de Salud Carlos III. Madrid, Spain

^5^Neurology Department, Parc Taulí Sabadell, Hospital Universitari, Universitat Autònoma de Barcelona, Sabadell, Barcelona, Spain

^6^Critical Care Department, Parc Taulí Sabadell, Hospital Universitari, Universitat Autònoma de Barcelona, Sabadell, Barcelona, Spain

^7^BSICOS Group, 13A, University of Zaragoza&CIBER-BBN Zaragoza, Spain

Table of Contents:

1. Methods

a. Design of neurocognitive stimulation software

b. Design of stimulation exercises

c. Neurocognitive intervention protocol

d. Patients' satisfaction survey

e. ICU personnel acceptance survey

2. Results

a. Results of patients' satisfaction survey

b. Results of ICU personnel acceptance survey

3. Tables

a. Table S1. Results of satisfaction survey

b. Table S2. Results of acceptance survey

4. Figures

a. Figure S1. Diagram of sessions planning based on alertness and mobility parameters

5. Videos

6. References

**1. Methods**

**a. Design of neurocognitive stimulation software**

The neurocognitive stimulation software was designed to present patients with an enjoyable and relaxing virtual environment consisting of 4 scenarios (wheat field, beach, forest, and mountain landscape) that patients can “walk” through. During sessions, a virtual co-therapist avatar –called AVA- accompanies patients. There are two different *entry* modes, one for the first neurocognitive session and another for subsequent sessions. The first session introduces AVA to patients, explains the purpose and procedure of sessions, provides time orientation and begins low neurocognitive stimulation. In subsequent sessions, AVA begins stimulation sessions by asking patients how they are and orientating them in time. During sessions, AVA delivers instructions to patients, motivates them to complete exercises, and encourages them to rest and relax. The software also includes two different *exit* modes. The *normal exit* mode is used when an entire session is completed; it is followed by a final screen where AVA informs patient that session has finished and says goodbye until the next session. The *forced exit* mode is used in situations in which a patient’s clinical deterioration requires urgent intervention by clinical staff.

The route through the virtual environment is initially predefined; however, therapists can reconfigure the route, modify the order in which cognitive exercises appear, or jump from one exercise to another using a series of software commands. In the virtual environment, lighting is linked to the computer’s internal clock time, so depending on the time of day in which the session occurs, it is possible to walk through the environment at dawn or dusk, in broad daylight, or even at night.

**b. Design of stimulation exercises**

The virtual environment includes different exercises focusing on attention and executive functions designed by our clinical team starting from exercises that have proven effective in previous cognitive rehabilitation programs [1]:

*-Guided-observation exercises*. These exercises have been especially designed for patients with problems with alertness and mobility. These exercises aim to maintain a minimum of attentional level. The virtual avatar guides patients through the virtual environment and asks them simple questions. Sometimes, therapists ask patients about the elements appearing on the screen and ask them to carry out simple actions, such as naming elements, naming colors, counting the number of a specific stimulus, recognizing elements of the environment, etc. Guided-observation exercises are also used to transition between exercises, allowing patients rest and relax.

*-Passive exercises*. These exercises require low levels of alertness and low mobility. Patients are asked to move their hand in specific periods to elicit a reaction in the virtual environment, such as make elements and sounds come out on or disappear from the screen.

*-Selective attention exercises*. These exercises require a moderate level of cognitive engagement. Patients are asked to move their hand to select specific stimuli based on certain internal characteristics. The exercises include progressively more difficult series that require patients have to focus on an increasing number of internal characteristics.

*-Working memory exercises*. These exercises aim to stimulate complex cognitive functions and therefore require greater cognitive effort. Patients are asked to complete geometric figures by moving their hand through sequencing and working memory abilities in progressively more complex series.

**c. Neurocognitive intervention protocol**

Before starting each neurocognitive session, we assessed patients’ alertness with the RASS score and their ability to raise autonomously each arm separately with their elbow straight against gravity. These two parameters determined the intensity of stimulation and which exercises to include in each session (Figure S1). In cognitively intact patients with muscle weakness or myopathy affecting their ability to maintain their arms high, the therapist and/or ICU nurse held up patients’ arms as needed to help them through each exercise.

Patients were asked to follow the avatar’s instructions and to use their hands to perform the stimulation exercises while they were bedridden. These sessions aimed to provide neurocognitive stimulation and engagement through the exercises (not necessarily obtaining correct answers), so therapists could provide cues and hints when patients answered wrong. Delirium did not preclude session participation if patients were able to follow the therapist’s commands.

**d. Healthy volunteers' and patients' satisfaction survey**

Before this proof-of-concept study, 6 healthy volunteers tested the platform in a session simulating ICU patients’ situation. Volunteers rated their experiences on a 5-point Likert scale (0=lowest, 5=highest) in a survey including 18 items in 6 categories: *comfort* (4 items), *physical fatigue* (3 items), *relaxation* (3 items), *boredom* (4 items), *system interaction* (4 items), and *overall satisfaction* (1 item). The first 6 patients included in the study also completed the ad hoc survey about their experience using the ENRIC Platform. Mean scores on the satisfaction survey were computed from the individual raw scores for all items included in each category after converting scores of negatively worded items to positives. Mean scores were analyzed using the Mann-Whitney U test.

**e. ICU personnel acceptance survey**

After finishing this proof-of-concept study, ICU personnel, including nurses, physicians, and physiotherapists, were surveyed to discover their opinions about the feasibility and compatibility of the ENRIC Platform with ICU workload. ICU personnel rated their opinions on a 5-point Likert scale (0=lowest or nothing, 5=highest or much) in an ad hoc survey including 11 items in 6 categories: *compatibility with pharmacologic treatment* (1 item), *with physiotherapy* (1 item), *with nursing practice* (1 item), *with physical infrastructure and ICU facilities* (3 items), *benefits for patients and acceptance* (4 items), and *feasibility of implementation in routine practice* (1 item). Finally, mean scores on the acceptance survey were computed from the individual raw scores for all items included in each category after converting scores of negatively worded items to positives.

**2. Results**

**a. Results of patients' satisfaction survey**

Table S1 compares satisfaction survey scores between healthy volunteers and critically ill patients. Patients scored higher than volunteers for *relaxation* (*p*=0.002) and *system interaction* (*p*=0.004) and lower for *boredom* (*p*=0.041).

**b. Results of ICU personnel acceptance survey**

Table S2 reports the global mean scores on the acceptance survey for ICU personnel and mean scores for physicians, nurses, and physiotherapists. *Compatibility with physical infrastructure and ICU facilities* (3.94/5) was the lowest-rated item for ICU professionals and *Compatibility with pharmacologic treatment* (4.72/5) and *compatibility with physiotherapy* (4.94/5) were the highest rated items.

**3. Tables**

**a. Table S1. Satisfaction survey scores in healthy volunteers versus critically ill patients**

**b. Table S2. Acceptance survey scores in physicians, nurses, and physiotherapists**

**4. Figures**

**Figure S1.** Diagram of sessions planning based on alertness and mobility parameters

RASS score and the ability to raise autonomously each arm separately with their elbow straight against gravity determined the intensity of stimulation and which exercises to include in each session

**5. Videos**

**Video 1.** Neurocognitive stimulation session with mechanically ventilated patient

**Video 2.** Details of neurocognitive stimulation software and system interaction

**6. References**

1. [Bogdanova Y](https://www.ncbi.nlm.nih.gov/pubmed/?term=Bogdanova Y%5BAuthor%5D&cauthor=true&cauthor_uid=26709580), [Yee MK](https://www.ncbi.nlm.nih.gov/pubmed/?term=Yee MK%5BAuthor%5D&cauthor=true&cauthor_uid=26709580), [Ho VT](https://www.ncbi.nlm.nih.gov/pubmed/?term=Ho VT%5BAuthor%5D&cauthor=true&cauthor_uid=26709580), [Cicerone KD](https://www.ncbi.nlm.nih.gov/pubmed/?term=Cicerone KD%5BAuthor%5D&cauthor=true&cauthor_uid=26709580). Computerized cognitive rehabilitation of attention and executive function in acquired brain injury: a systematic review. J Head Trauma Rehabil. 2016;31:419-33.

**Table S1.** Satisfaction survey scores in healthy volunteers versus critically ill patients

|  | | | |
| --- | --- | --- | --- |
|  | **Healthy volunteers**  **(n=6)** | **Critically ill patients**  **(n=6)** | ***p*** |
| Comfort, M (SD) | 4.25 (0.45) | 4.75 (0.42) | 0.07 |
| Physical fatigue, M (SD) | 0.78 (0.75) | 1.00 (0.99) | 0.82 |
| Boredom, M (SD) | 0.96 (0.58) | 0.33 (0.82) | 0.041 |
| Relaxation, M (SD) | 4.11 (0.46) | 4.95 (0.13) | 0.002 |
| System interaction, M (SD) | 3.67 (0.61) | 4.83 (0.30) | 0.004 |
| Overall satisfaction, M (SD) | 4.33 (0.52) | 4.83 (0.41) | 0.18 |
| M, mean; SD, standard deviation  *p*-values for non-parametric Mann-Whitney U test | | | |

**Table S2.** Acceptance survey scores in physicians, nurses and physiotherapist

|  | **Physicians** | **Nurses** | **Physiotherapist** | **Total** |
| --- | --- | --- | --- | --- |
|  | **(n=8)** | **(n=9)** | **(n=1)** | **(n=18)** |
| Pharmacologic treatment compatibility, M (SD) | 4.75 (0.46) | 4.67 (0.50) | 5.00 (0.00) | 4.72 (0.46) |
| Physiotherapy compatibility, M (SD) | 5.00 (0.00) | 4.89 (0.33) | 5.00 (0.00) | 4.94 (0.24) |
| Nursing practice compatibility, M (SD) | 3.88 (0.35) | 4.67 (0.50) | 5.00 (0.00) | 4.33 (0.59) |
| Patients benefits and acceptance, M (SD) | 4.29 (0.34) | 4.56 (0.35) | 3.67 (0.00) | 4.39 (0.39) |
| Compatibility with physical infrastructure and ICU facilities, M (SD) | 3.96 (0.28) | 4.00 (0.25) | 3.33 (0.00) | 3.94 (0.29) |
| Feasibility for implementing in routine practice M (SD) | 4.25 (0.46) | 4.88 (0.35) | 5.00 (0.00) | 4.59 (0.51) |
| M, mean; SD, standard deviation | | | | |
